# Blind Source Separation Based on Double-Mutant Butterfly Optimization Algorithm

**DOI:** 10.3390/s22113979

**Published:** 2022-05-24

**Authors:** Qingyu Xia, Yuanming Ding, Ran Zhang, Minti Liu, Huiting Zhang, Xiaoqi Dong

**Affiliations:** 1 Communication and Network Laboratory, Dalian University, Dalian 116622, China; xiaqingyu0315@163.com (Q.X.); nancy444@163.com (R.Z.); hwilting@163.com (H.Z.); dongxiaoqi3360@163.com (X.D.); 2 National Laboratory of Radar Signal Processing, Xidian University, Xi’an 710071, China; liuminti@163.com

**Keywords:** blind source separation, independent component analysis, butterfly optimization algorithm, dynamic transformation probability, population reconstruction mechanism, differential evolution operator, sine cosine operator

## Abstract

The conventional blind source separation independent component analysis method has the problem of low-separation performance. In addition, the basic butterfly optimization algorithm has the problem of insufficient search capability. In order to solve the above problems, an independent component analysis method based on the double-mutant butterfly optimization algorithm (DMBOA) is proposed in this paper. The proposed method employs the kurtosis of the signal as the objective function. By optimizing the objective function, blind source separation of the signals is realized. Based on the original butterfly optimization algorithm, DMBOA introduces dynamic transformation probability and population reconstruction mechanisms to coordinate global and local search, and when the optimization stagnates, the population is reconstructed to increase diversity and avoid falling into local optimization. The differential evolution operator is introduced to mutate at the global position update, and the sine cosine operator is introduced to mutate at the local position update, hence, enhancing the local search capability of the algorithm. To begin, 12 classical benchmark test problems were selected to evaluate the effectiveness of DMBOA. The results reveal that DMBOA outperformed the other benchmark algorithms. Following that, DMBOA was utilized for the blind source separation of mixed image and speech signals. The simulation results show that the DMBOA can realize the blind source separation of an observed signal successfully and achieve higher separation performance than the compared algorithms.

## 1. Introduction

Blind source separation (BSS), sometimes referred to as blind signal processing, is capable of recovering a source signal from an observed signal in the absence of critical information, such as source and channel [1,2,3]. Due to its high adaptability and other advantages, BSS has been employed in a variety of research fields in recent years, such as image processing, medical evaluation, radar analysis, speech recognition, and machinery [4,5,6,7,8].

Independent component analysis (ICA) is an important BSS method [9]. However, the conventional natural gradient algorithm (NGA) is too reliant on gradient information [10], whereas the fast fixed-point algorithm for ICA (FastICA) is sensitive to the initial solution [11]. Thus, improving the speed and precision with which the separation matrix is solved and obtaining higher-quality separated signals have significant practical implications.

To address the aforementioned issues, a swarm intelligence algorithm with a solid coevolution mechanism is gradually applied to ICA. Preliminary research indicates that BSS based on a swarm intelligence algorithm outperforms traditional BSS methods in terms of separation performance [12]. Li et al. [13] utilized the improved particle swarm optimization (PSO) for ICA. The disadvantage is the poor search capability of PSO in the later stages of iteration. Wang et al. [14] employed the improved artificial bee colony (ABC) optimization as the optimization algorithm for ICA, despite the fact that this optimization algorithm is very parameter dependent. Luo et al. [15,16] applied the improved fireworks algorithm (FA) to the radar signal processing, while the fireworks algorithm is prone to local extremum. Wen et al. [17] used a genetic algorithm (GA) to ICA, although the local search capability of GA is limited.

The butterfly optimization algorithm (BOA) was developed in 2018. It was inspired by the behavior of butterflies looking for food and demonstrated high robustness and global convergence while addressing complex optimization problems [18]. According to preliminary studies, BOA is very competitive in function optimization when compared to other metaheuristic algorithms, such as ABC, cuckoo search algorithm (CSA), firefly algorithm (FA), GA, and PSO [19]. It does, however, face several difficulties. For instance, it is possible to fall into local optimization when dealing with high-dimensional complexity prior to optimization operation. Additionally, inappropriate parameters result in a slow convergence speed of BOA. Therefore, scholars have proposed a series of improved algorithms to improve the performance of BOA. Arora et al. [20] combined BOA and ABC, enhancing the algorithm’s exploitation capacity. Long et al. [21] provided a pinhole image learning strategy based on the optical principle that can help avoid premature convergence in the algorithm. Fan et al. [22] introduced a new fragrance coefficient and a different iteration and update strategy. Mortazavi et al. [23] proposed a novel fuzzy decision strategy and introduced a notion of “virtual butterfly” to enhance the search capability of BOA. Zhang et al. [24] proposed a heuristic initialization strategy combined with greedy strategy, which improved the diversity of the initial population. Li et al. [25] introduced weight factor and Cauchy mutation to BOA, enhancing the ability of the algorithm to jump out of local optimization. The above references are some improvement methods of BOA. Although they can improve the search performance of the algorithm to some extent and reduce the premature convergence phenomenon in the algorithm, most improved algorithms only focus on the improvement of single search performance and ignore the balance between global search ability and local search ability.

Based on the foregoing research, and in response to the limitations of the low separation performance of conventional ICA methods and the lack of search ability in basic BOA, this paper presents an ICA method based on the double-mutant butterfly algorithm (DMBOA). Firstly, the dynamic transformation probability and population reconstruction mechanisms are introduced to assist the algorithm in maintaining its search balance and increasing its capacity to avoid the local optimum. The differential evolution operator is then introduced in the global position update to allow for mutation, while the sine cosine operator is introduced in the local position update to allow for mutation, hence, enhancing the algorithm’s exploitation capacity. Finally, the superiority of DMBOA is verified in benchmark function and BSS problem.

To summarize, the major contributions of this paper are given as follows:

(1) An ICA method based on DMBOA is designed to address the low-separation performance of conventional ICA. DMBOA is used to optimize the separation matrix W, maximize the kurtosis, and finally, complete the separation of observation signals.
(2) Three improved strategies are designed for the insufficient search capability of the basic BOA, which coordinate the global search and local search of the algorithm while improving BOA searching ability.
(3) Simulation results show that DMBOA outperforms the other nine algorithms when optimizing 12 benchmark functions. In the BSS problem, DMBOA is capable of successfully separating mixed signals and achieving higher separation performance than the compared algorithms.

The remainder of this paper is organized as follows: Section 2 introduces the basic theory of BSS. Section 3 discusses the details of the BOA. Section 4 addresses the DMBOA implementation. Section 5 provides simulation analysis, which verifies the effectiveness of the proposed algorithm. Section 6 concludes the paper and summarizes the major contributions.

The main literature contributions in the introduction are introduced in Table 1.

## 2. Basic Theory of Blind Source Separation

### 2.1. Linear Mixed Blind Source Separation Model

The linear mixed BSS model is described below:

(1) X(t)=AS(t)+N(t)

where *t* is the sampling moment, *A* is a mixed matrix of order m×n (m≥n), *X*(*t*) is a vector of the m-dimensional observed signals, $X(t)=[X_{1}(t),X_{2}(t),\ldots ,X_{m}(t)]$, *S*(*t*) is a vector of the *n*-dimensional source signals, $S(t)=[S_{1}(t),S_{2}(t),\ldots ,S_{n}(t)]$, *N*(*t*) is a vector of the *m*-dimensional noise signals. BSS represents the cases in which an optimization algorithm determines the separation matrix, *W*, when only the observed signals, *X*(*t*), are known. In such instances, the separated signals, *Y*(*t*), are obtained using Equation (2).

(2) Y(t)=WX(t)

where $Y(t)=[Y_{1}(t),Y_{2}(t),\ldots ,Y_{n}(t)]$.

To ensure the feasibility of BSS, the following assumptions are required:

(1) The mixing matrix, A, should be reversible or full rank, and the number of observed signals should be larger than or equal to the number of source signals (i.e.,).
(2) From a statistical standpoint, each source signal is independent of the others, and at most, one signal follows a Gaussian distribution, because multiple Gaussian processes remain a Gaussian process after mixing and, hence, cannot be separated.

Due to the lack of source signal and channel information, it is difficult to discern the signal’s amplitude and order following BSS, a phenomenon known as fuzziness. Although BSS is fuzzy, its fuzziness has a negligible effect on the results in the majority of scientific research and production practices. 

**Figure 1:**
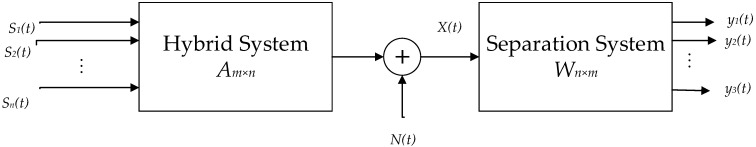
Linear mixed blind source separation model.

Figure 1 shows the linear mixed blind source separation model.

### 2.2. Signal Preprocesing

Prior to performing BSS on observed signals, it is usually essential to preprocess the signals in order to simplify the separation process. De-averaging and whitening are two widely used preprocessing techniques.

The de-averaging processing method is shown in Equation (3).

(3) X=X−E(X)

The purpose of whitening is to eliminate the signals’ correlation. The whitening operation in BSS is used to remove the second-order correlations between signals in space, ensuring that the observed signals received by the sensor remain uncorrelated in space and simplifying the algorithm complexity. The signal, *V*, after whitening is expressed as follows:

(4) $$V=QX=E^{-1/2}U^{T}X$$

where *Q* is a whitening matrix, *U* is a characteristic matrix composed of eigenvectors corresponding to the *n* maximum eigenvalues of the autocorrelation matrix, $R_{XX}=E[XX^{H}]$, of the observed matrix, *X*, and $E=diag(d_{1},d_{2},\ldots d_{n})$ is a diagonal matrix composed of these eigenvalues.

The separation matrix, W, is an orthogonal matrix, which can be expressed as the product of a series of rotation matrices [26]. Taking three source signals as an example, the separation matrix, W, is defined as follows:

(5) $$W=\left[\begin{array}{ccc} 1 & 0 & 0\\ 0 & \cos\theta_{1} & \sin\theta_{1}\\ 0 & \sin\theta_{1} & \cos\theta \end{array}\right]•\left[\begin{array}{ccc} \cos\theta_{2} & 0 & -\sin\theta_{2}\\ 0 & 1 & 0\\ \sin\theta_{2} & 0 & \cos\theta_{2} \end{array}\right]•\left[\begin{array}{ccc} \cos\theta_{3} & -\sin\theta_{3} & 0\\ \sin\theta_{3} & \cos\theta_{3} & 0\\ 0 & 0 & 1 \end{array}\right]$$

### 2.3. Separation Principle

When performing BSS on mixed signals using ICA, it is necessary to first select an appropriate criterion for determining the statistical independence of the separated signals. Afterwards, the objective function is established and optimized using the appropriate algorithm. This leads to the separation matrix with the strongest independence of the separated signals.

The commonly used independence criterion of signals includes mutual information, kurtosis, and negative entropy. Kurtosis is calculated using Equation (6) as follows:

(6) $$K(y_{i})=kurt(y_{i})=E\{y_{i}^{4}\}-3(E{\left\{y_{i}^{2}\right\}}^{2})$$

where *y_i_* is a gaussian random variable.

The sum of absolute values of kurtosis is used as a criterion of signal independence in this paper, and the objective function is specified as follows:

(7) $$fit_{i}=\frac{1}{\sum_{i=1}^{n}\left|K(y_{i})\right|+\varepsilon }$$

where ε is an extremely small amount that prevents division by zero. According to the information theory, for a gaussian random vector *y_i_*, when $E[yy^{T}]=I$, the larger the kurtosis of the signals, the greater their independence. The above-mentioned DMBOA will be used to optimize the separation matrix *W*, to maximize the kurtosis, and finally complete the separation of the observed signals.

## 3. Butterfly Optimization Algorithm (BOA)

BOA is an optimization technique inspired by the foraging behavior of butterflies. Each butterfly in BOA serves as a search operator and performs the optimization process in the search space. Butterflies are capable of perceiving and distinguishing between different fragrance intensities, and each butterfly emits a fragrance of a certain intensity. Assume that the intensity of the fragrance produced by butterflies is proportional to their fitness; that is, as butterflies move from one location to another, their fitness will change accordingly. When a butterfly detects the fragrance of another, it will move toward the butterfly with the strongest fragrance. This stage is referred to as “global search.” On the contrary, if the butterfly is unable to perceive the fragrance of other butterflies, it will move randomly. This stage is referred to as “local search.” The global and local searches are switched during the search process by switching the probability *p*.

The fragrance can be formulated as follows:

(8) $$f=cI^{a}$$

where *f* is the perceived intensity of the fragrance, i.e., the fragrance’s intensity as perceived by other butterflies, *c* is the sensory modality, *I* is the stimulus intensity, depending on fitness, and *a* is the mode-dependent power exponent, which accounts for the various degrees of absorption, a∈[0,1]. The value of *c* is updated by Equation (9) as follows:

(9) $$c^{t+1}=c^{t}+0.025/(c^{t}\times T)$$

where *t* and *T* represent the current and maximum number of iterations, respectively.

When butterflies sense the stronger fragrance in the area, they move towards the strongest one. This stage is calculated as follows:

(10) $$x_{i}^{t+1}=x_{i}^{t}+(r^{2}\times g-x_{i}^{t})\times f$$

When a butterfly is unable to perceive the surrounding fragrance, it moves randomly. This stage is calculated as follows:

(11) $$x_{i}^{t+1}=x_{i}^{t}+(r^{2}\times x_{j}^{t}-x_{k}^{t})\times f$$

where $x_{i}^{t}$ represents the position of butterfly individual *i* in generation *t*, $x_{j}^{t}$ denotes the position of butterfly individual *j* in generation *t*, $x_{k}^{t}$ indicates the position of butterfly individual *k* in generation *t*, *r* shows a random number between 0 and 1, and *g* stands for the gl obal optimal position.

The pseudo code of BOA is provided in Algorithm 1.
**Algorithm 1:BOA**Input: Objective function *f*(x), butterfly population size *N*, stimulation concentration *I*, sensory modality c=0.01, power exponent a=0.1, conversion probability p=0.8, Maximum number of iterations *T*.1. Initialize population2. **While**
*t* < *T* 3.   **for**
*i* = 1: *N*4.   Calculate fragrance using Equation (8) 5.   Generate a random number *rand* in [0, 1] 6.   **if**  *rand* < *p* 7.     Update position using Equation (10) 8.   **else** 9.     Update position using Equation (11) 10.    **end if** 11.    **if** $f(x_{i}^{t})\geq f(g)$ 12.      $g=x_{i}^{t}$, $f(g)=f(x_{i}^{t})$ 13.    **end if** 14.    Update the value of *c* using Equation (9) 15. **end for** 16. **end while** 17. Output the global optimal solution


## 4. Double-Mutant Butterfly Optimization Algorithm (DMBOA)

### 4.1. Dynamic Transition Probability

Local and global searches are controlled in the basic BOA by the constant switching probability *p*, which implies that during the iterative process of the algorithm, BOA will allocate 80% of its search capability to global search and 20% to local search. In this search mode, about 80% of the butterflies in the population will be attracted to the best butterfly, *g*. Therefore, if the best butterfly, *g*, falls into the local optimum, it will strongly guide other butterflies to this unpopular position in the search space, making it more difficult for the algorithm to avoid the local extreme value, so it converges prematurely.

A reasonable search process should begin with a strong global search in the early stages of the algorithm, quickly locate the scope of the global optimal solution in the search space, and appropriately enhance the local development capability in the latter stages of the exploration, all of which contribute to the optimization accuracy of the algorithm. The dynamic switching probability, *p*_2_, is proposed in this paper to balance the proportions of local and global search to achieve a more effective optimization strategy. The dynamic conversion probability, *p*_2_, is shown in Equation (12).

(12) $$p_{2}=0.8-0.3\times \sin(\frac{\pi }{\mu }{\left(\frac{t}{T}\right)}^{2})$$

where μ takes constant 2.

**Figure 2:**
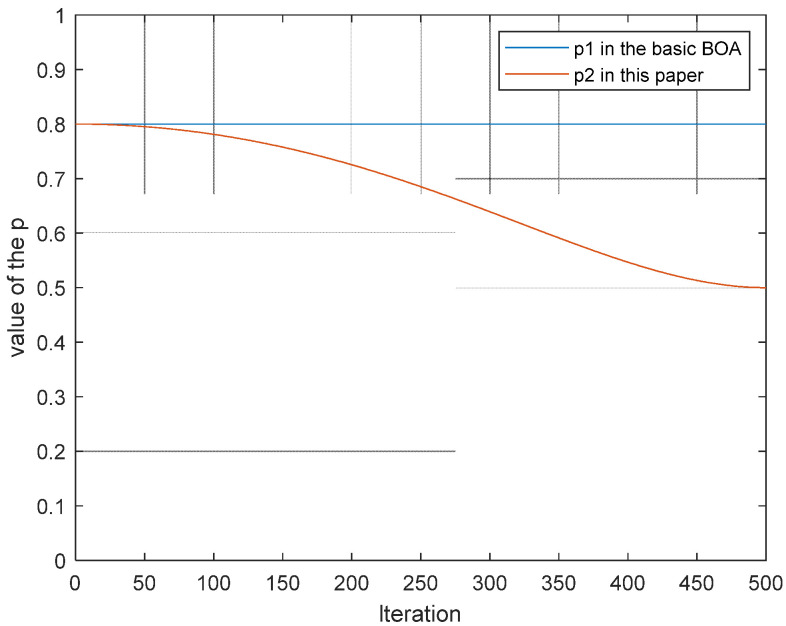
Iterative curve of transformation probability *p*.

As seen in Figure 2, the dynamic conversion probability, *p*_2_, proposed in this paper, gradually converges to 0.5 as iteration progresses. It can strike a balance between global search in the early stages and local development in the latter stages.

### 4.2. Improvement in Update Function

When some butterflies move completely at will or when a large number of butterflies congregate at non-global extreme points, the convergence speed of BOA is significantly slowed and falls into local extreme values. Two mutation operators, the differential evolution [27,28] and sine cosine operator [29], are used in this paper to improve BOA.

The differential evolution operator utilizes three-parameter variables for global search, which results in a faster convergence rate and simplifies the process of obtaining the global optimal value, which is why it is used for global search. The sine cosine operator possesses the periodicity and oscillation of the sine cosine function, which enables it to avoid falling into the local extremum, accelerate the convergence speed of the algorithm, and be applied to local search.

The global search variation is expressed as follows:

(13) $$x_{i}^{t+1}=x_{i}^{t}\times r_{1}+F\times [\lambda \times (g-x_{j}^{t})-(1-\lambda )\times (g-x_{k}^{t})]$$

The local search variation is determined as follows:

(14) $$x_{i}^{t+1}=\left\{ \begin{array}{l} x_{i}^{t}\times \sin r_{2}+(1-r_{1})\times {|r}_{3}\times g-x_{i}^{t}|r_{4}<0.5\\ x_{i}^{t}\times \cos r_{2}+(1-r_{1})\times {|r}_{3}\times g-x_{i}^{t}|r_{4}\geq 0.5 \end{array}\right.$$

where the mutation operator, F∈[0,2], is a real constant factor, $r_{2}$ is a random number with a value range between 0 and 2π, and λ and *r*_3_ are random numbers with a value range between 0 and 1. The parameter *r*_1_ is calculated as follows:

(15) $$r_{1}=\delta -t\times \frac{\delta }{T}$$

where δ takes constant 2.

### 4.3. Population Reconstruction Mechanism

The counter *count* is introduced, with an initial value of 0. If the global optimal solution, *g*, remains constant, the *count* increases by 1. If the global optimal solution, *g*, changes, the counter is reset. When the *count* is greater than or equal to 0.1∗T, the default optimization stops. To preserve previous optimization results and increase the population diversity to avoid local optimums, 20% of the individuals, including the optimal solution, are randomly selected from the original population, while the remaining 80% of individuals are discarded and replaced with new randomly generated individuals.

**Figure 3:**
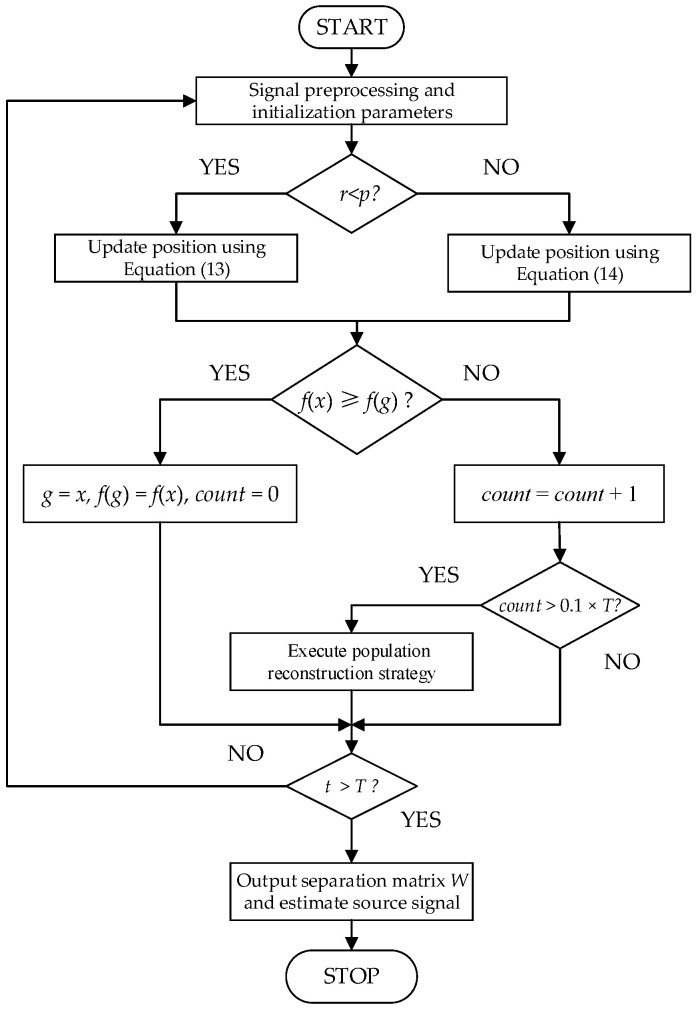
The flow chart of DMBOA-ICA.

Algorithm 2 gives the pseudo code of DMBOA, and Figure 3 shows the flow chart of DMBOA-ICA.
**Algorithm 2:DMBOA**Input: Objective function *f*(x), butterfly population size *N*, stimulation concentration *I*, sensory modality c=0.01, power exponent a=0.1, maximum number of iterations *T*. counter count=0.1. Initialize population2. **While***t* < *T* 3.   **for**
*i* = 1: *N*4.    Calculate fragrance using Equation (8) 5.    Calculate conversion probability *p* using Equation (8) 6.    Generate a random numbers *rand* in [0, 1] 7.      **if**  *rand* < *p* 8.      Update position using Equation (13) 9.     **else** 10.       Update position using Equation (14) 11.   **end if**
12.   **if**
$f(x_{i}^{t})\geq f(g)$ 13.     $g=x_{i}^{t}$, $f(g)=f(x_{i}^{t})$, count=0
14.   **else** 15.**        **count=count+1 16.   **end if**
17.   **if**
count≥0.1∗T 18.       Execute population reconstruction strategy 19.   **end if**
20.   Update the value of *c* using Equation (9) 21.   **end for** 22. **end while** 23. Output the global optimal solution


The DMBOA proposed in this paper enhances the basic BOA in three aspects. Firstly, the dynamic transformation probability coordination algorithm is implemented using both local and global search. The double-mutant operator is then incorporated into the algorithm update function to enhance the local search capability of the algorithm. Finally, a population reconstruction mechanism is introduced to avoid falling into local optimums in the event of optimization stagnation. Through the above three improvement methods, DMBOA can effectively overcome the poor search capability of the basic BOA, which makes it easy to fall into local optimums. However, when compared to the basic BOA, DMBOA has a higher computational complexity, as each iteration of DMBOA requires calculating the value of the calculator *count* and reconstructing the population when it falls into optimization stagnation, which, in turn, increases the calculations required by this algorithm.

## 5. Simulation and Result Analysis

### 5.1. Evalution of DMBOA on Benchmark Function

To more accurately and comprehensively verify the efficacy of DMBOA, 12 test functions were used with varying characteristics for experiments. The detailed characteristics of each test function are listed in Table 2. It features four single-mode test functions (*F*_1_*–F*_4_), as well as eight multi-mode test functions (*F*_5_*–F*_12_). In Table 2, *Dim* denotes the function dimension, *Scope* represents the value range of *x*, and *f*_min_ indicates the ideal value of each function. There is only one global optimal solution for single-mode test functions and no local optimal solution. They are suitable for evaluating the local development capability of the algorithm. On the contrary, there are many local optimal solutions for multimodal test functions. Numerous algorithms that perform well with low modal functions perform poorly with high modal functions and are prone to local optimization or oscillation between local extrema. The high-modal test function is usually used to evaluate the global search capability of the algorithm [30].

DMBOA is compared against nine algorithms in the experiment, namely GWO [31], WOA [32], CF-AW-PSO [33], HPSOBOA [34], FPSBOA [35], BOA [18], BOA_1 (dynamic conversion probability), BOA_2 (introduce double-mutant operator), and BOA_3 (introduce population reconstruction mechanism). For all ten algorithms, the population size *N* = 30 and the total number of iterations *T* = 500. The parameters of DMBOA are shown in Algorithm 2, while the parameters of other algorithms are shown in references [31,32,33,34,35]. Table 2 shows the optimal fitness value (BEST), the average fitness value (MEAN), the standard deviation (STD), and the running time (TIME), tested by 10 algorithms, such as DMBOA under 12 test functions in Table 2, in which the time unit is seconds. The test results of DMBOA have been bold in Table 3. Each algorithm was performed separately 30 times to minimize the error, and all experiments were conducted on a laptop equipped with an Intel (R) Core (TM) i7-6500 CPU at 2.50 GHz and 8 GB of RAM.

As shown in Table 3, DMBOA is capable of obtaining the optimal values for these 12 test functions, and the optimal values for each function are closer to *f*_min_ in Table 2. The search accuracy of BOA_1, BOA_2, and BOA_3 proposed in this paper is also better than the original BOA, demonstrating the efficacy of the three improvement strategies utilized in this paper. DMBOA has a higher search accuracy than the improved algorithm with a single strategy, indicating that under the joint influence of different strategies, the optimization ability and stability of the algorithm are improved to the greatest extent. Overall, the test results of BOA_2 are closer to those of DMBOA. The STD of data can reflect the degree of dispersion. According to the test results in Table 3, DMBOA has the smallest STD for each test function, indicating that it is more robust and stable than the compared algorithms when dealing with both low- and high-modal problems. As for the calculation time in Table 3, DMBOA has a medium execution time. According to the data in the table, the test time for DMBOA under the five test functions of *F*_2_, *F*_4_, *F*_5_, *F*_11_, and *F*_12_ is less than that of the original BOA. This indicates that, although the time complexity of DMBOA is higher in theory than that of the original BOA, the high convergence accuracy of DMBOA enables it to find the global optimal solution more quickly, particularly for the two test functions, *F*_11_ and *F*_12_.

**Figure 4:**
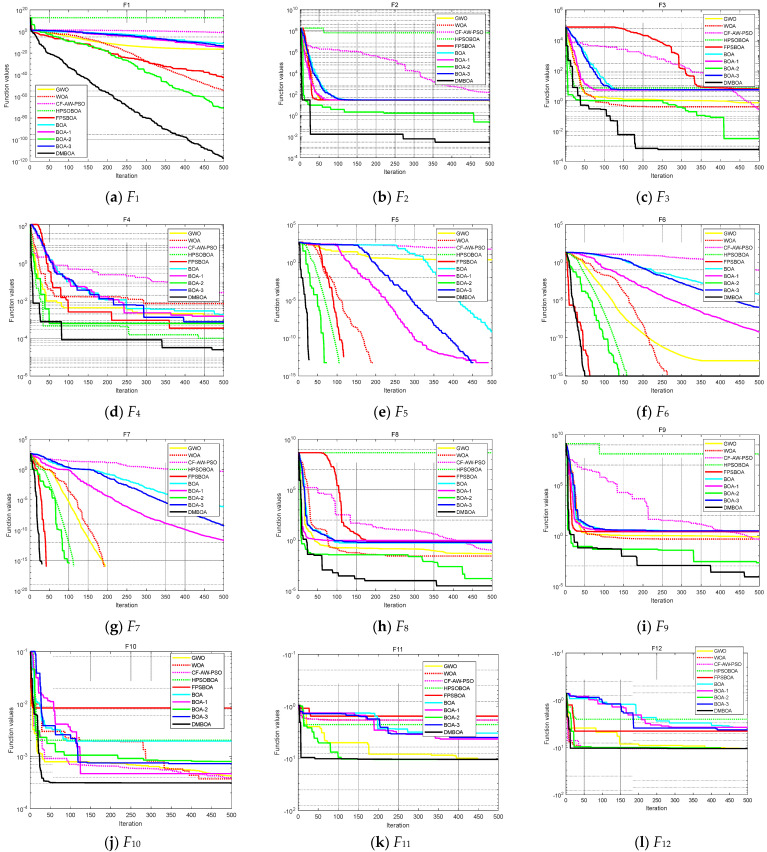
Convergence curves of 10 algorithms on 12 test function in Table 2.

Figure 4 depicts the iteration history of the ten algorithms tested on the 12 test functions in Table 2. As seen in Figure 4, the DMBOA developed in this study has the fastest iteration speed and maximum convergence accuracy among all the convergence history graphs. This demonstrates that, when compared to other algorithms, DMBOA is capable of obtaining the optimal solution in the shortest amount of time. BOA-1, BOA-2, and BOA-3, which are improved by a single strategy, improved convergence speed and optimization accuracy to a certain extent when compared to basic BOA, indicating that each strategy performed satisfactorily and effectively, but not as well as the DMBOA, which is improved by a hybrid strategy. The feasibility of the three improved strategies is further verified. GWO can be iterated until it reaches the theoretical optimal value under *F*_5_ and *F*_7_. The overall convergence performance of WOA is general. The convergence speed of CF-AW-PSO is slow in the early stages. The iteration results of HPSOBOA under F_1_, *F*_2_, *F*_6_, and *F*_7_ are poor. FPSBOA outperforms *F*_5_, *F*_6_, and *F*_7_ in terms of convergence curve and search performance.

### 5.2. Speech Signal Separation

**Figure 5:**
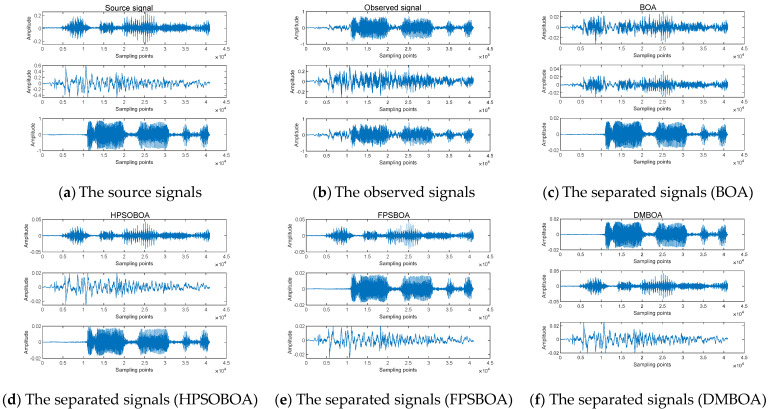
Effect drawing of speech signal separation. (**a**) The waveform of source signals; (**b**) the waveform of observed signals; (**c**) The waveform of BOA separated signals; (**d**) The waveform of HPSOBOA separated signals; (**e**) The waveform of FPSBOA separated signals; (**f**) The waveform of DMBOA separated signals.

Three speech signals are used as the source signals, which are then mixed to obtain the observed signals. To acquire the separated signals, DMBOA, BOA, HPSOBOA, and FPSBOA are used to blindly separate the observed signals. The simulation diagram is depicted in Figure 5. The sampling frequency and sampling point of voice signals are 40,964 and 1000, respectively.

In order to quantitatively analyze and compare the separation performance of the four algorithms, the time, similarity coefficient, performance index (PI), and PESQ [36] are employed in this study. The data are shown in Table 4 with a time unit of seconds.

The PESQ metric is based on the wide-band version recommended in ITU-T [37], and its range is extended from −0.5 to 4.5. The higher its value, the better the quality of the speech signal. The similarity coefficient and PI are expressed in Equations (16) and (17) as follows:

(16) $$\rho_{ij}=\frac{\left|\sum_{i=1}^{N}s_{i}(t)y_{j}(t)\right|}{\sqrt{\sum_{i=1}^{N}s_{i}^{2}(t)\sum_{t=1}^{N}y_{j}^{2}(t)}}$$

(17) $$PI=\frac{1}{N(N-1)}\sum_{i=1}^{N}\{(\sum_{i=1}^{N}\frac{\left|G_{ij}\right|}{\max_{i}|G_{il}|}-1)+(\sum_{j=1}^{N}(\frac{\left|G_{ij}\right|}{\max_{j}|G_{il}|}-1)\}$$

In Equation (16), $\rho_{ij}$ is a similarity index used to compare the source signal with the separated signal. The greater the $\rho_{ij}$, the more effective the separation. In this section, $\rho_{ij}$ is a 3×3 matrix. The maximum value of each channel is taken as the experimental data, and *N* is set to 3. Additionally, in Equation (17), 

; the closer the *PI* is to 0, the more similar the separated signal is to the source signal.

In comparison to Figure 5, the separated signals have a different amplitude and order than the source signals, indicating the fuzziness of BSS. The signals separated by BOA are partially distorted. The signals separated by HPSOBOA and FPSBOA are partially deformed. The signals separated by DMBOA are highly consistent with the waveform of the source signal and have a strong separation effect.

As shown in Table 4, DMBOA produces not only the highest similarity coefficient and PESQ but also the smallest PI of the separated signal, allowing for a more accurate restoration of the source signal. Moreover, the operation time of DMBOA is shorter than that of the examined algorithms.

### 5.3. Image Signal Separation

**Figure 6:**
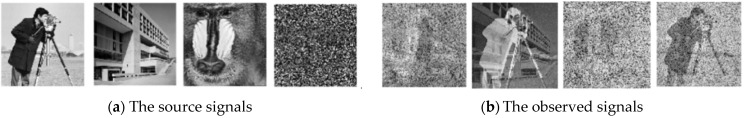

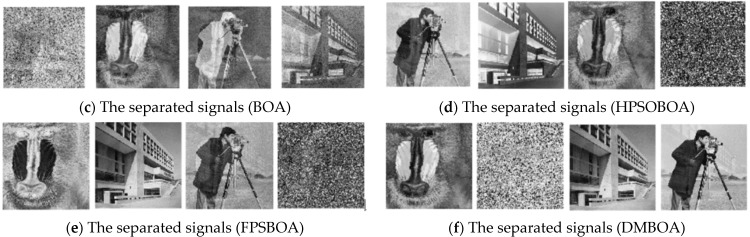
Effect drawing of image signal separation. (**a**) The image of source signals; (**b**) The image of observed signals; (**c**) The image of BOA separated signals; (**d**) The image of HPSOBOA separated signals; (**e**) The image of FPSBOA separated signals; (**f**) The image of DMBOA separated signals.

Three gray-scale images and one random noise image are used as source signals, and they are combined to produce the observed signals. To acquire the separated signals, DMBOA, BOA, HPSOBOA, and FPSBOA are used to blindly separate the observed signals. In this section, *N* is assumed to be 4, and the pixels of the image are 256×256; $\rho_{ij}$ is a 4×4 matrix. Figure 6 illustrates the simulation result and Table 5 compares the similarity coefficient, PI, and duration of separated signals, as well as the SSIM [38] of the output image. The SSIM proves to be a better error metric for comparing the image quality with better structure preservation. They are in the range of [0, 1], which is a value closer to one indicating better structure preservation:

(18) $$\mathrm{SSIM}=\frac{\left(2\mu_{\hat{x}}\mu_{x}+C_{1}\right)\left(2\sigma_{\hat{x}x}+C_{2}\right)}{\left(\mu_{\hat{x}}^{2}+\mu_{x}^{2}+C_{1}\right)\left(\sigma_{\hat{x}}^{2}+\sigma_{x}^{2}+C_{2}\right)}$$

where $C_{1}$ and $C_{2}$ are constant, $\sigma_{\hat{x}x}$ represents the covariance of image, $\mu_{\hat{x}}$ and $\mu_{x}$ represent the mean value of the two images, respectively, $\sigma_{\hat{x}}$ and $\sigma_{x}$ represent the variance in the two images, respectively.

As seen in Figure 6, the images separated by DMBOA are similar to the source images, but the images separated by other algorithms have varying degrees of ambiguity. Additionally, as demonstrated by the data in Table 5, the separation performance of DMBOA is superior to that of the examined algorithms.

## 6. Conclusions

This paper proposed a novel double-mutant butterfly optimization algorithm (DMBOA), which is a major improvement on the butterfly optimization algorithm (BOA) and applied to blind source separation (BBS). The algorithm incorporates a double-mutant operator and a population reconstruction mechanism, which enhances the capability of local development and avoids local optimization. The proposed technique was initially explored and further developed through the use of a dynamic conversion probability balancing method. The following conclusions are drawn from the simulation results:

(1) When optimizing 12 benchmark functions (four low-modal and eight high-modal), DMBOA outperforms the other nine algorithms. The three improvement methods proposed in this study increased the performance of BOA to varying degrees in the algorithm ablation experiment. All of this demonstrates that DMBOA has a high level of search performance and strong robustness.
(2) DMBOA outperforms the other algorithms in the BSS and is capable of successfully separating the mixed speech and image signals.

## Figures and Tables

**Table 1:** The main literature contributions.

Algorithm Type	Name	Method	Conclusion	Reference
Conventional ICA	NGA	Based on gradient information	The separation performance of conventional algorithms is low and need to be further improved.	Amari [10]
	FastICA	Based on fixed point iteration		Barros et al. [11]
Intelligent optimization ICA	PSO-ICA	Introduce PSO into ICA	Introducing swarm intelligence algorithms into ICA improves the separation performance compared with conventional ICA. But there are problems with these swarm intelligence algorithms.	Li et al. [13]
	ABC-ICA	Introduce ABC into ICA		Wang et al. [14]
	FA-ICA	Introduce FA into ICA		Luo et al. [15,16]
	GA-ICA	Introduce GA into ICA		Wen et al. [17]
Improved algorithms of BOA	BOA/ABC	Combines BOA and ABC	Most improved algorithms only improve the single search performance of BOA, but ignore the balance between global search ability and local search ability.	Arora et al. [20]
	PIL-BOA	Provides a pinhole image learning strategy based on the optical principle		Long et al. [21]
	SABOA	Introduces a new fragrance coefficient and a different iteration strategy		Fan et al. [22]
	FBOA	Proposes a novel fuzzy decision strategy and introduces a notion of “virtual butterfly”		Mortazavi et al. [23]
	OEbBOA	Proposes a heuristic initialization strategy combined with greedy strategy		Zhang et al. [24]
	IBOA	Introduces weight factor and Cauchy mutation		Li et al. [25]

**Table 2:** Basic information of benchmark functions.

Function	*Dim*	*Scope*	*f*_min_
$F_{1}(x)=\sum_{i=1}^{n}\left|x_{i}\right|+\prod_{i=1}^{n}{|x}_{i}|$	30	[–10, 10]	0
$F_{2}(x)=\sum_{i=1}^{n}\left[100{\left(x_{i+1}-x_{i}^{2}\right)}^{2}+{\left(x_{i}-1\right)}^{2}\right]$	30	[−30, 30]	0
$F_{3}(x)=\sum_{i=1}^{n}{\left(\left[x_{i}+0.5\right]\right)}^{2}$	30	[−100, 100]	0
$F_{4}(x)=\sum_{i=1}^{n}ix_{i}^{4}+\mathrm{random}[0,1)$	30	[−1.28, 1.28]	0
$F_{5}(x)=\sum_{i=1}^{n}\left[x_{i}^{2}-10\cos\left(2\pi x_{i}+10\right)\right]$	30	[−5.12, 5.12]	0
$F_{6}(x)=-20\exp\left(-0.2\sqrt{\frac{1}{n}\sum_{i=1}^{n}x_{i}^{2}}\right)-\exp\left(\frac{1}{n}\sum_{i=1}^{n}\cos(2\pi x_{i})\right)+20+e$	30	[−32, 32]	0
$F_{7}(x)=\frac{1}{4000}\sum_{i=1}^{n}x_{i}^{2}-\prod_{i=1}^{n}\cos\left(\frac{x_{i}}{\sqrt{i}}\right)+1$	30	[−600, 600]	0
$F_{8}(x)=\frac{\pi }{n}\left\{10\sin\left(\pi y_{1}\right)+{\sum_{i=1}^{n}\left(y_{i}-1\right)}^{2}\left[1+10\sin^{2}\left(\pi y_{i+1}\right)\right]+{\left(y_{n}-1\right)}^{2}\right\}+\sum_{i=1}^{n}u\left(x_{i},10,100,4\right),y_{i}=1+\frac{x_{i}+1}{4}u\left(x_{i},a,k,m\right)=\{\begin{array}{l} k{\left(x_{i}-a\right)}^{m}x_{i}>a\\ 0-a<x_{i}<a\\ k{\left(-x_{i}-a\right)}^{m}x_{i}<-a \end{array}$	30	[−50, 50]	0
$F_{9}(x)=0.1\left\{\sin^{2}\left(3\pi x_{i}\right)+\sum_{i=1}^{n}{\left(x_{i}-1\right)}^{2}\left[1+\sin^{2}\left(3\pi x_{i}+1\right)\right]+{\left(x_{n}-1\right)}^{2}\left[1+\sin^{2}\left(2\pi x_{n}\right)\right]\right\}+\sum_{i=1}^{n}u\left(x_{i},5,100,4\right)$	30	[−50,50]	0
$F_{10}(x)=\sum_{i=1}^{11}\left[ai-\frac{x1\left(bi^{2}+bix2\right)}{bi^{2}+bix3+x4}\right]$	4	[−5, 5]	0.00030
$F_{11}(x)=-\sum_{i=1}^{7}{\left[\left(X-a_{i}\right){\left(X-a_{i}\right)}^{T}+c_{i}\right]}^{-1}$	4	[0, 10]	−10.4028
$F_{12}(x)=-\sum_{i=1}^{10}{\left[\left(X-a_{i}\right){\left(X-a_{i}\right)}^{T}+c_{i}\right]}^{-1}$	4	[0, 10]	−10.5363

**Table 3:** Comparative analysis of performance of 10 swarm intelligence algorithms.

Function	Index	DMBOA	BOA	BOA_1	BOA_2	BOA_3	HPSOBOA	FPSBOA	GWO	WOA	CF_AW_PSO
F1	BEST	**1.28 × 10^−119^**	2.75 × 10^−11^	2.42 × 10^−14^	1.13 × 10^−62^	3.01 × 10^−13^	3.19 × 10^11^	9.73 × 10^−51^	1.20 × 10^−16^	9.40 × 10^−53^	0.12256
	MEAN	**2.04 × 10^5^**	9.01 × 10^7^	3.51 × 10^7^	8.52 × 10^5^	4.71 × 10^7^	4.58 × 10^13^	2.43 × 10^8^	7.29 × 10^8^	8.05 × 10^9^	1.40 × 10^12^
	STD	**2.47 × 10^5^**	2.01 × 10^9^	7.47 × 10^7^	7.85 × 10^7^	7.85 × 10^8^	1.61 × 10^14^	1.68 × 10^9^	1.63 × 10^9^	8.84 × 10^9^	2.04 × 10^12^
	TIME	**0.1610**	0.1527	0.1543	0.1771	0.1732	0.1757	0.1375	0.1927	0.0901	1.0270
F2	BEST	**1.06 × 10^−2^**	28.9471	28.8818	0.1786	28.0715	2.41 × 10^8^	2.89 × 10^1^	26.8769	27.6766	2.03 × 10^2^
	MEAN	**1.24 × 10^5^**	2.91 × 10^6^	2.51 × 10^6^	1.39 × 10^6^	2.47 × 10^6^	2.44 × 10^8^	1.32 × 10^6^	1.89 × 10^6^	1.97 × 10^6^	1.62 × 10^6^
	STD	**1.63 × 10^6^**	2.67 × 10^7^	1.68 × 10^7^	1.68 × 10^7^	2.04 × 10^7^	9.84 × 10^6^	1.58 × 10^7^	1.80 × 10^7^	1.93 × 10^7^	1.32 × 10^7^
	TIME	**0.1941**	0.2077	0.1844	0.1844	0.2290	0.1947	0.1884	0.2050	0.0794	0.9862
F3	BEST	**1.28 × 10^−3^**	5.1259	4.738	0.0098	4.9992	6.3584	4.8811	0.6259	0.4128	0.3316
	MEAN	**2.87 × 10^2^**	2.32 × 10^3^	2.01 × 10^3^	3.30 × 10^2^	2.04 × 10^3^	2.66 × 10^2^	2.91 × 10^3^	6.50 × 10^2^	6.24 × 10^2^	1.94 × 10^3^
	STD	**4.02 × 10^3^**	8.84 × 10^3^	7.43 × 10^3^	4.48 × 10^3^	9.00 × 10^3^	3.46 × 10^3^	7.78 × 10^3^	4.68 × 10^3^	5.10 × 10^3^	4.42 × 10^3^
	TIME	**0.1356**	0.1241	0.1316	0.1478	0.1524	0.1369	0.1279	0.1774	0.0631	0.9525
F4	BEST	**7.93 × 10^−5^**	0.0020	8.33 × 10^−4^	6.09 × 10^−4^	1.30 × 10^−3^	1.09 × 10^−4^	5.31 × 10^−4^	1.44 × 10^−3^	0.0049	0.0485
	MEAN	**0.4327**	3.4488	2.3577	1.2125	1.4712	0.5487	3.6951	0.7935	1.0180	0.9897
	STD	**5.1646**	14.7580	11.7394	10.0441	14.9266	5.9768	15.277	7.2482	8.1546	7.1023
	TIME	**0.3257**	0.3312	0.3017	0.3247	0.3436	0.3058	0.3163	0.2940	0.1530	1.0780
F5	BEST	**0**	2.85 × 10^−10^	0	0	0	0	0	0.7624	0	47.5728
	MEAN	**2.3163**	1.04 × 10^2^	33.2498	4.5992	90.2747	10.9330	1.87 × 10^2^	26.5955	27.2819	1.59 × 10^2^
	STD	**27.8726**	1.20 × 10^2^	88.8153	38.8205	1.21 × 10^2^	52.0126	8.82 × 10^1^	67.5291	72.5040	75.2091
	TIME	**0.1925**	0.1992	0.1797	0.1795	0.2186	0.1676	0.1645	0.1920	0.0734	0.9903
F6	BEST	**8.88 × 10^−16^**	4.74 × 10^−5^	3.24 × 10^−7^	8.88 × 10^−16^	1.21 × 10^−6^	8.88 × 10^−16^	8.88 × 10^−16^	1.22 × 10^−13^	6.57 × 10^−15^	0.8873
	MEAN	**0.1272**	3.4204	2.2722	0.2123	3.4058	0.6122	0.1782	0.7996	0.6367	7.2342
	STD	**1.4421**	6.1540	5.1366	1.6815	6.2388	2.8252	2.1388	3.1655	2.7333	4.3789
	TIME	**0.1650**	0.1561	0.1485	0.2010	0.1724	0.1481	0.1467	0.1926	0.0730	1.0238
F7	BEST	**0**	3.70 × 10^−7^	8.08 × 10^−11^	0	6.84 × 10^−9^	0	0.3697	0.0033	0	0.5772
	MEAN	**2.9803**	27.5437	17.7892	4.9745	22.8844	9.8251	3.2284	6.1030	6.1503	19.5304
	STD	**39.4556**	97.3967	78.3987	45.4127	95.8473	51.8421	40.1433	44.5673	47.6451	40.2275
	TIME	**0.1864**	0.1834	0.1744	0.1475	0.1956	0.1674	0.1836	0.2231	0.0904	0.9302
F8	BEST	**6.45 × 10^−5^**	0.5278	0.6101	3.39 × 10^−4^	0.5155	1.42 × 10^8^	5.57 × 10^5^	0.0438	0.0262	0.1533
	MEAN	**7.93 × 10^5^**	4.05 × 10^6^	1.86 × 10^6^	1.81 × 10^6^	3.66 × 10^6^	2.22 × 10^8^	9.69 × 10^7^	3.38 × 10^6^	3.84 × 10^6^	1.65 × 10^6^
	STD	**2.26 × 10^7^**	3.96 × 10^7^	2.83 × 10^7^	2.96 × 10^7^	2.83 × 10^7^	1.32 × 10^8^	1.24 × 10^8^	3.55 × 10^7^	3.99 × 10^7^	2.58 × 10^7^
	TIME	**0.6626**	0.6407	0.6175	0.6476	0.6813	0.6804	0.6465	0.4189	0.3076	1.1649
F9	BEST	**3.00 × 10^−5^**	2.8907	2.8577	6.88 × 10^−4^	2.9815	6.61 × 10^8^	2.5389	0.6075	0.3928	0.8492
	MEAN	**4.84 × 10^6^**	8.96 × 10^6^	4.97 × 10^6^	4.92 × 10^6^	8.55 × 10^6^	7.11 × 10^8^	3.09 × 10^7^	7.28 × 10^6^	8.05 × 10^6^	5.20 × 10^6^
	STD	**4.01 × 10^7^**	8.44 × 10^7^	6.45 × 10^7^	6.45 × 10^7^	7.98 × 10^7^	1.35 × 10^8^	1.40 × 10^8^	7.66 × 10^7^	8.25 × 10^7^	5.42 × 10^7^
	TIME	**0.6237**	0.6170	0.6374	0.6372	0.6380	0.6234	0.6133	0.4383	0.3051	1.1549
F10	BEST	**3.29 × 10^−4^**	4.63 × 10^−4^	7.52 × 10^−4^	0.0024	6.95 × 10^−4^	8.33 × 10^−3^	1.21 × 10^−2^	3.62 × 10^−4^	0.0011	3.31 × 10^−4^
	MEAN	**0.0014**	0.0108	0.0087	0.0031	0.0075	0.0250	0.0139	0.0193	0.0125	0.0132
	STD	**0.0092**	0.0440	0.0352	0.0174	0.0397	0.0266	0.0182	0.0130	0.0137	0.0149
	TIME	**0.1415**	0.1331	0.1256	0.1441	0.1470	0.1204	0.1351	0.1030	0.0566	0.8962
F11	BEST	**−10.4021**	−3.7065	−4.2248	−10.3921	−4.3732	−2.7479	−6.4141	−10.3998	−7.2097	−7.8124
	MEAN	**−10.0248**	−3.0691	−3.9299	−9.8669	−3.2063	−2.5950	−4.5366	−7.6326	−5.9612	−6.9726
	STD	**1.1633**	1.7467	1.4404	1.3712	1.4478	1.2843	1.1177	2.3504	1.7862	1.0625
	TIME	**0.2247**	0.4710	0.4779	0.2023	0.5053	0.5345	0.1971	0.1303	0.0934	0.8702
F12	BEST	**−10.5398**	−4.2295	−4.5870	−10.4547	−4.4975	−2.6101	−5.1456	−10.5191	−5.2541	−7.3815
	MEAN	**−9.9728**	−2.8359	−2.8770	−9.4217	−3.1161	−2.5639	−3.8055	−8.0916	−5.0373	−6.7461
	STD	**0.5395**	1.3041	1.1196	1.9452	1.3458	1.2225	1.0012	2.1849	0.7045	1.3569
	TIME	**0.2381**	0.5797	0.5812	0.2390	0.5975	0.6086	0.2210	0.1440	0.1157	0.8930

**Table 4:** Data of speech signal separation performance evaluation index.

Algorithm	BOA	HPSOBOA	FPSBOA	DMBOA
similarity coefficient	0.8584	0.9001	0.9741	0.9877
	0.7951	0.9274	0.9526	0.9927
	0.8560	0.9432	0.9363	0.9763
PI	0.3054	0.2041	0.1687	0.1329
time	35.78	26.14	25.41	22.48
PESQ	2.06	2.23	2.30	2.44

**Table 5:** Data of image signal separation performance evaluation index.

Algorithm	BOA	HPSOBOA	FPSBOA	DMBOA
similarity coefficient	0.81190 85460 87570 8378	0.88780 90210 90740 9253	0.97840 95520 93010 9222	0.99820 99070 98740 9833
PI	0.2601	0.1986	0.1524	0.1163
time	37.91	34.25	30.51	26.74
SSIM	0.8340	0.9015	0.9282	0.9647

## Data Availability

Not applicable.
